# A Rare Case of Intraperitoneal Desmoplastic Fibroblastoma

**DOI:** 10.7759/cureus.22134

**Published:** 2022-02-11

**Authors:** Gabriella Savage, Joanna Perry-Keene, David Grieve

**Affiliations:** 1 Department of Surgery, Sunshine Coast Hospital and Health Service, Sunshine Coast, AUS; 2 Department of Pathology and Laboratory Medicine, Sunshine Coast Hospital and Health Service, Sunshine Coast, AUS; 3 School of Medicine, Griffith University, Sunshine Coast, AUS

**Keywords:** benign tumour, myofibroblastic lesion, fibroblastic lesion, soft tissue lesions, desmoplastic fibroblastoma

## Abstract

Desmoplastic fibroblastoma is a benign soft tissue tumor of indolent nature. It is more prevalent in males in their fourth to sixth decades of life and typically presents in the upper extremities, feet, and back. Other, uncommon locations have been reported as well, including the oral cavity and retroperitoneum. Histological examination demonstrates bland spindle cells in a dense collagenous stroma. The tumor neither recurs nor metastasizes. In this report, we discuss a case of a female patient who presented with symptoms concerning for intra-abdominal sepsis and was subsequently diagnosed with an intraperitoneal desmoplastic fibroblastoma. There is no evidence that this condition has been previously reported in the literature in the English language. The lesion was excised during laparoscopy and the patient showed no evidence of recurrence on magnetic resonance elastography (MRE) imaging 12 months later.

## Introduction

Desmoplastic fibroblastoma is a rare, benign soft tissue lesion of indolent nature; it presents as a well-circumscribed, occasionally lobulated firm mass macroscopically resembling cartilage [1]. The lesion is more prevalent in males in their fourth to sixth decades of life and usually presents in the upper extremities, feet, and back. More uncommon locations have also been reported and include the oral cavity and heart [2,3]. Differential diagnoses for the condition include fibroblastic/myofibroblastic lesions [1]. Histological examination demonstrates bland-looking spindled, reactive-appearing fibroblasts in a highly collagenous stroma [4,5]. The tumor does not recur. We present a case of a 33-year-old female who underwent a laparoscopic excision of a desmoplastic fibroblastoma lesion from the gastrocolic ligament.

## Case presentation

A 33-year-old female presented to the hospital with central abdominal pain, fever, and tachycardia. The pain had been of sudden onset and stabbing in nature. It was constant and exacerbated by movement. The pain was not migratory, nor had the patient experienced it before. She was not menstruating and had no previous history of ovarian pathology. There was no history of loss of appetite, nausea, vomiting, or weight loss. The only surgical history was of an emergency lower-segment cesarean section (LSCS) 12 months prior to this presentation for the delivery of a healthy preterm female baby. There was no relevant medication or family history of note. She was a non-smoker and did not drink alcohol or take illicit drugs. Physical examination demonstrated a fever of 38.3 °C and a heart rate of 102 beats per minute. Her abdomen was soft, with supraumbilical tenderness. There were no other abnormal examination findings.

**Figure 1:**
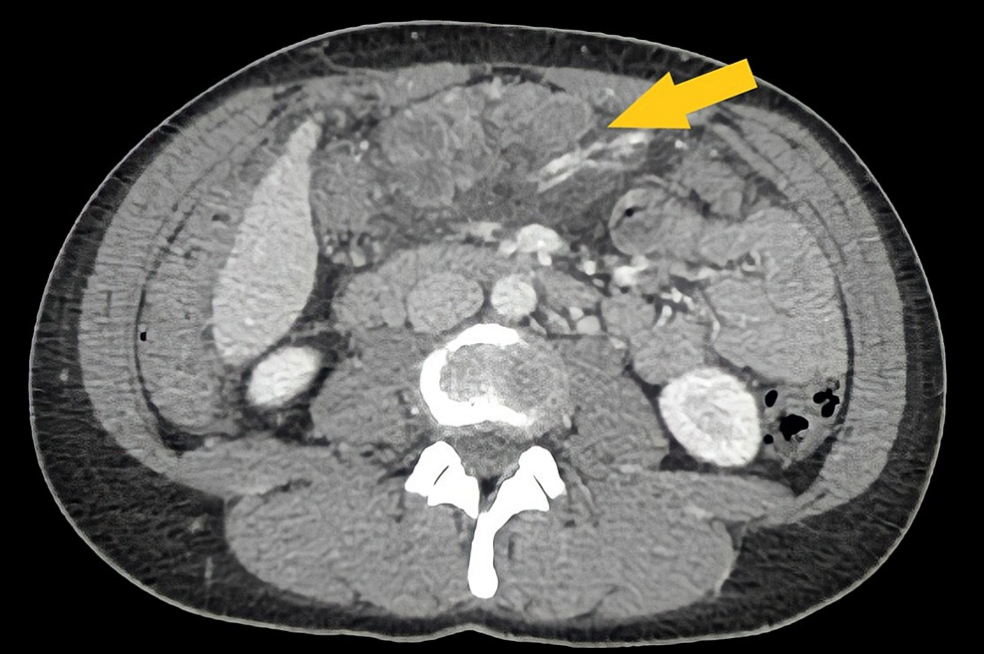
CT abdomen: axial plane, portal venous phase. The arrow indicates epigastric lesion CT: computed tomography

Complete blood count, urea and electrolytes, and liver function tests were normal. C-reactive protein on admission was 41 mg/L. A CT scan of the abdomen demonstrated a complex mass located inferior to the gastric antrum and a moderate amount of free fluid within the abdomen and pelvis (Figure 1). The mass had ill-defined margins. The provisional diagnosis at this stage was localized viscus perforation with associated intraperitoneal abscess. The patient was started on empirical intravenous ampicillin (2 g every four hours), gentamicin (320 mg daily), and metronidazole (500 mg three times per day) and admitted overnight for observation and further evaluation.

**Figure 2:**
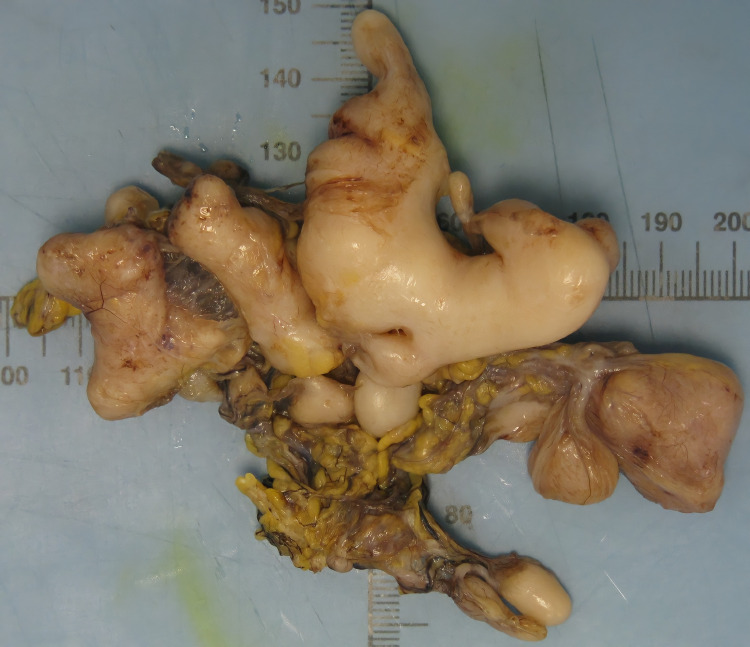
Macroscopic specimen: gross examination showed a yellow to gray lobulated mass with a firm consistency

**Figure 3:**
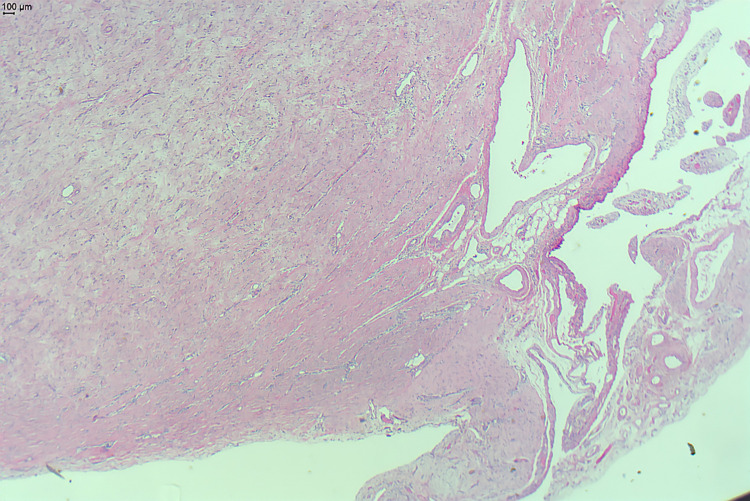
Histopathological features: lobulated edge, bland spindle cells in a dense collagenous stroma

Magnetic resonance enterography (MRE) was performed, which demonstrated a T2 hypodense, non-specific serpiginous lesion inferior to the pylorus. Differential diagnoses based on the MRE included a duplication cyst or cystic sarcomatous lesion. There was a symptomatic improvement in her condition within 24 hours, and antibiotics were de-escalated. A laparoscopy was performed, and the lesion was found to be associated with the gastrocolic ligament and was excised in toto for histology (Figures 2, 3).

Gross examination of the specimen showed a lobulated mass of yellow to grey color with a firm consistency. The histopathology demonstrated bland-looking spindled fibroblast cell proliferation, raising the possibility that this was a variant of the hyperplastic scar. Immunohistochemical workup showed weakly positive cytoplasmic staining for B-catenin and negative for CD34 and desmin. The CD34 stain, used in the diagnosis of some gastrointestinal stromal tumors, was negative. VSP6 Flat showed no translocation excluding nodular fasciitis. Other stains including S-100, used in the diagnosis of nerve sheath tumors, and CD-68, a marker of histiocytic origin, were negative. It was concluded that the lesion represented benign fibrous proliferation favoring desmoplastic fibroblastoma.

## Discussion

Desmoplastic fibroblastoma is associated with classic histopathologic features including paucicellular, bland stellate-spindled, reactive-appearing fibroblasts, and myofibroblastic cells within a highly collagenous myxoid stroma [1,4]. The tumor cells are typically positive for vimentin, confirming mesenchymal origin. They have a variable positivity for alpha-smooth muscle actin, which is used to identify smooth muscle cells and myofibroblasts in reactive or neoplastic tissue. There is no immunoreactivity documented for desmin, CD34, and S-100 [1,4]. Reactivity for these markers may indicate sarcomatous, nerve sheath, and other mesenchymal origins. Features of desmoplastic fibroblastoma on MRI include medium signal intensity on T1-weighted imaging and low signal intensity on T2-weighted images [5].

In 1995, Evans described desmoplastic fibroblastoma as a benign tumor of subcutaneous or muscle tissue [6]. This tumor is more prevalent in males with a peak incidence in the fourth to sixth decades of life; desmoplastic fibroblastoma characteristically presents as a slow-growing non-tender mass of indolent nature [1,6]. Recent literature suggests that desmoplastic fibroblastoma encompasses a broader heterogeneous group of tumors than what was originally thought [4]. Resection may not always be necessary if imaging characteristics and biopsy confirm a benign desmoplastic fibroblastoma lesion.

In the literature, case reports on desmoplastic fibroblastoma are confined to the limbs or on the back. Fibroblastic or myofibroblastic lesions have a similar appearance; however, they can be differentiated from desmoplastic fibroblastoma by their poorly circumscribed nature, more cellular and fascicular patterns, and prominent vasculature. Other rare locations where the lesion has been reported include the oral cavity, the ribs, and mediastinum [2,3,7]. To the best of our knowledge, this is the first reported case of intraperitoneal desmoplastic fibroblastoma. Features on MRI are suggestive and require clinical correlation [5]. There is no evidence in the literature that a history of LSCS (Pfannenstiel incision) is related to the diagnosis of desmoplastic fibroblastoma. It is unclear why the patient presented with a fever and tachycardia; however, extensive investigation and multi-disciplinary input did not reveal an infective cause, and the diagnostic uncertainly warranted laparoscopic investigation.

There have been no reported recurrences in the literature following excision of desmoplastic fibroblastoma in other more common locations. In this case, an MRE at three months was normal. Further review with repeat MRE at 12 months was also normal.

## Conclusions

Desmoplastic fibroblastoma is a benign tumor that usually presents in the upper extremities and back of adult men. Owing to its low prevalence, there is scarce literature on the guidance of its management. This case report highlights an unusual presentation of this particular tumor and emphasizes the importance of both thorough preoperative planning and detailed histological examination to exclude similar tumors that may offer malignant potential.
